# Hospital Meetings

**Published:** 1905-12-23

**Authors:** 


### HOSPITAL MEETINGS.

### ROYAL ORTHOPÆDIC HOSPITAL.

There was a very small attendance at the annual Court of Governors of this hospital, held on Wednesday, December 13.

The chair was taken by the President of the hospital, the Earl of Denbigh, who, in moving the adoption of the report for 1904, said they had little to report as a separate hospital, as they had been working in conjunction with the National Orthopædic Hospital. The rent they were now receiving for the site of the old premises did not figure in the report, as they had only begun to enjoy it at the beginning of this year, their income would, therefore, be henceforward increased by £1,400. The amalgamation with the National Orthopædic Hospital having now been satisfactorily carried out, they would probably have to raise capital for rebuilding by means of a mortgage. The successful conclusion of their amalgamation scheme had been received with special gratification by the trustees of the King Edward's Hospital Fund, as showing that a start was being made in their policy for amalgamating the special hospitals of London.

The committee of management having been re-elected and other business transacted, the annual court became a special one, in order to approve certain technicalities in connection with the disposal of the site, and the amalgamation.

### THE SURGICAL AID SOCIETY.

The 43rd annual meeting of the Surgical Aid Society was held at 4 p.m. at the Mansion House, on Wednesday, the 13th instant, the Lord Mayor being in the chair.

The annual report and statement of accounts were taken as read, everyone present being supplied with a printed copy.

The Chairman made a short speech, expressing his conviction of the extreme value of the work done by the society, and the increasing scope for its efforts, as testified by the enormous number of applications for tickets received by all subscribers.

The Bishop of Croydon explained how much he had seen of the working of the society during the many years of his life as a parish priest.

Sir W. Treloar proposed a vote of thanks, which was carried unanimously, to Mr. Samuel Watson for his services as treasurer, and a request that he would allow himself to be re-elected. No one had done more for the society than Mr. Watson, who had been a subscriber for 35 years.

Proceedings terminated with a vote of thanks to the Lord Mayor for presiding, and a request that he would become a vice-president of the society.

